# Barriers and facilitators for men to attend prenatal care and obtain HIV voluntary counseling and testing in Brazil

**DOI:** 10.1371/journal.pone.0175505

**Published:** 2017-04-17

**Authors:** Nava Yeganeh, Mariana Simon, Deborah Mindry, Karin Nielsen-Saines, Maria Cristina Chaves, Breno Santos, Marineide Melo, Brenna Mendoza, Pamina Gorbach

**Affiliations:** 1 Dept of Pediatrics, Division of Infectious Diseases, David Geffen School of Medicine at UCLA, Los Angeles, CA, United States; 2 Grupo Hospitalar Conceicao, Servico de Infectologia Hospital Nossa Senhora da Conceicao/GHC, Porto Alegre, RS Brazil; 3 UCLA Center for Culture and Health, Department of Psychiatry and Behavioral Sciences, NPI-Semel Institute for Neuroscience, Los Angeles, CA, United States; 4 Department of Epidemiology, Fielding School of Public Health at UCLA, Los Angeles, CA United States; University of Washington, UNITED STATES

## Abstract

**Background:**

Providing HIV voluntary counseling and testing (VCT) to men who attend their partner's prenatal care is an intervention with potential to reduce HIV transmission to women and infants during the vulnerable period of pregnancy. Little is known about the acceptability of this intervention in global settings outside of Africa.

**Methods:**

We conducted in-depth qualitative interviews to evaluate potential barriers and facilitators to prenatal care attendance for HIV VCT with 20 men who did and 15 men who did not attend prenatal care with their partners at Hospital Conceiçao in Porto Alegre, Brazil. Men were recruited at the labor and delivery unit at Hospital Conceiçao via a scripted invitation while visiting their newborn infant. Interviews lasted from 35–55 minutes and were conducted in Portuguese by a local resident trained extensively in qualitative methods. All interviews were transcribed verbatim, translated, and then analyzed using Atlast.ti software. An analysis of themes was then conducted using direct quotes and statements. We applied and adapted the AIDS Risk Reduction Theoretical Model and HIV Testing Decisions Model to the qualitative data to identify themes in the 35 interviews.

**Results:**

If offered HIV testing during prenatal care, all men in both groups stated they would accept this intervention. Yet, individual, relationship and systemic factors were identified that affect these Brazilian men's decision to attend prenatal care, informing our final conceptual model. The men interviewed had a general understanding of the value of HIV prevention of mother to child transmission. They also described open and communicative relationships with their significant others and displayed a high level of enthusiasm towards optimizing the health of their expanding family. The major barriers to attending prenatal care included perceived stigma against HIV infected individuals, men’s lack of involvement in planning of the pregnancy as well as inconvenient scheduling of prenatal care, due to conflicting work schedules.

**Conclusions:**

Brazilian men displayed high levels of HIV-related knowledge as well as open communication about HIV testing; especially when compared to findings from African studies. Future efforts should reorient prenatal care towards providing care to the entire family with a clear focus on protecting the infant from preventable diseases. Formally inviting men to prenatal care and providing them an acceptable medical excuse from work may enhance male involvement.

## Introduction

Engaging men in prenatal care has potential as a tool to protect pregnant women and infants from preventable infectious diseases. In traditional settings, gender roles confer power to men to make at least 75% of decisions related to women’s health issues.[1] In African countries, prenatal male involvement has been shown to improve acceptance of HIV testing in women, reduce negative outcomes of disclosure to partner, increase condom use, improve ART compliance and infant feeding strategies and decrease HIV infection in infants.[2–6] Testing and treating men for sexually transmitted infections (STIs) allows individuals to obtain treatment and adopt safer behaviors to decrease transmission. [2,7] However, most implementation efforts related to HIV prevention are directed towards women only.[8,9] As a result, men lack the crucial awareness of their role in improving and protecting the health of their family.[8–10] Studies performed in Cameroon, Malawi, Tanzania and South Africa suggest that men’s reluctance in attending antenatal care may be due to men identifying antenatal care as a woman’s domain and/or female space. Thus, men perceive that attending prenatal care is “unmanly.” [4,9,11–14] In some African countries, cultural norms dictate that it is unacceptable for a woman to “lead” or tell the husband what to do. [11,15] However, this has not been evaluated in other cultural contexts where HIV continues to be a problem, such as Latin America.

Porto Alegre is the capital and largest city in the Brazilian southern state of Rio Grande do Sul. Southern Brazil has the highest rate in the country of HIV/AIDS cases, AIDS related mortality, HIV infected pregnant women, as well as the highest rates of congenital syphilis and hepatitis C.[16–18] The AIDS prevalence rate in Rio Grande do Sul in 2015 is 34.7 cases/100,000, as compared to the national rate of 17.9 cases/100,000.[19,20]. At Hospital Conceiçao, a tertiary HIV referral center in the metropolitan city of Porto Alegre, Brazil: more than 95% of pregnant women receive prenatal care. However, between 2006–2013, of the 1,132 HIV-infected women who delivered, 371 (32.8%) of HIV-infected pregnant women were diagnosed with HIV at the time of labor and delivery (instead of during prenatal care), and 42 of this group seroconverted during pregnancy (i.e, had a prior negative HIV test earlier in gestation) resulting in higher than expected rates of HIV mother to child transmission.[21] In order to curb the incidence of acute HIV acquisition during pregnancy, health units providing prenatal care associated with Hospital Conceiçao are now encouraged to offer HIV voluntary counseling and testing (VCT) to male partners of HIV negative pregnant women.[22,23] In a study performed at this site in 2011, 54% of male partners attended prenatal care received HIV testing; however, most obtained testing during the last few months of pregnancy.[23] In the non-study setting, health care providers at the site stated that most male partners do not attend prenatal care. The most commonly given reasons for men’s non-testing *by women* included partner’s unavailability because of work and men’s low perceived risk for HIV infection.[23] Men have not yet been directly interviewed regarding uptake of HIV testing and participation during prenatal care. Therefore, we conducted a qualitative study of men with pregnant partners in prenatal care at Hospital Conceiçao in Porto Alegre, Brazil to identify barriers and facilitators affecting male partner attendance in prenatal care.

## Methods and materials

### Study site

Hospital Conceiçao is a 1200 bed, publicly funded hospital which serves as the major referral center for HIV-infected patients, including pregnant women, for the state of Rio Grande do Sul. Approximately 7,000 women per year receive prenatal care at its main campus or satellite clinics. As previously described by our other studies, most patients receiving care at Hospital Conceiçao are from the lower socio-economic strata as many middle, upper middle and upper class residents often receive care from the private sector.[22,23] It is estimated that among pregnant women 3–5% of those in prenatal care, and 5–10% of women without prenatal care in Rio Grande do Sul are HIV infected. [20,21,24,25] As of 2011, all satellite clinics have been encouraged to offer HIV VCT to male partners during women’s antenatal clinic visits.

Recruitment methods: Men visiting their partner and newborn in the post-partum unit of Conceiçao hospital were invited to participate in the study between March and July 2016. Men were included in the study if they were 18 years old or older, had a live born infant at Conceiçao hospital in the last week and have a wife/partner who participated in prenatal care at one of the affiliated health units. Purposive sampling was used to include more men who were non-white given research showing that this population is at higher risk for STIs [16,17] as well as lower rates of participation in partner testing.[17,23,26–28] Interviewer read an information sheet/verbal consent to each participant in Portuguese. Once the participant consented, interviewer signed the Information sheet/consent form and gave a copy to participant. This procedure received IRB approval from both UCLA and Hospital Conceiçao, as a written consent with the participant’s name would be the only identifying information linking him to our study. In-depth interviews were conducted by a female local resident of Porto Alegre in Portuguese. Interviewer is a certified therapist with prior experience in performing in depth interviews and she was extensively trained to perform these particular interviews by investigators. Participants volunteered for the interviews and were not compensated for their participation. The interviewer used a semi-structured interview guide adapted from a previously validated framework evaluating barriers and facilitators to accepting HIV testing [29], with questions probing factors affecting participation on an individual level (fear, stigma, perception of risk/benefit); in regards to relationship and fatherhood (intimacy, communication, trust, responsibility, support, gender); as well as systems issues (cost, convenience, masculinity, health-seeking behaviors). Interviews lasted from 35 to 55 minutes. Interviews were continued until we felt that consistent information was being relayed and that we could successfully fulfill our research goals, which occurred after interview 35.

### Analysis

The interviews were audiotaped, transcribed and translated into English. They were then coded using Atlas.ti software (GmbH, Berlin Germany) by two study investigators and discrepancies in coding were discussed and used to clarify standardized code definitions, with an intercoder reliability (ICR) of 0.82 (Supplemental Information: Partner Responses for Manuscript). Thematic data analysis included indexing the data through the application of topical codes. Initially, our analysis was focused on identifying specific barriers to HIV testing, but as our study evolved, our analysis emphasized factors that facilitated or impeded men’s attendance in prenatal care. Therefore, themes were analyzed for content separately by men’s prenatal care attendance (or lack of attendance) and also by race, given the racial disparities in health noted in other studies.[17,23,30] Since we were most interested in barriers and facilitators to attending prenatal care, our analysis drew on the data topically coded as “prenatal care good”, “prenatal care bad,” “motivation for testing” “improvements to testing” and “stigma”. These codes highlighted discussions regarding the interviewee’s perceptions of individual, relationship and systemic factors that interacted to motivate or prevent his participation in prenatal care. Direct verbatim quotes were chosen that helped support themes identified. The study was approved by the UCLA and Hospital Conceiçao institutional review boards.

## Results

**Fig 1:**
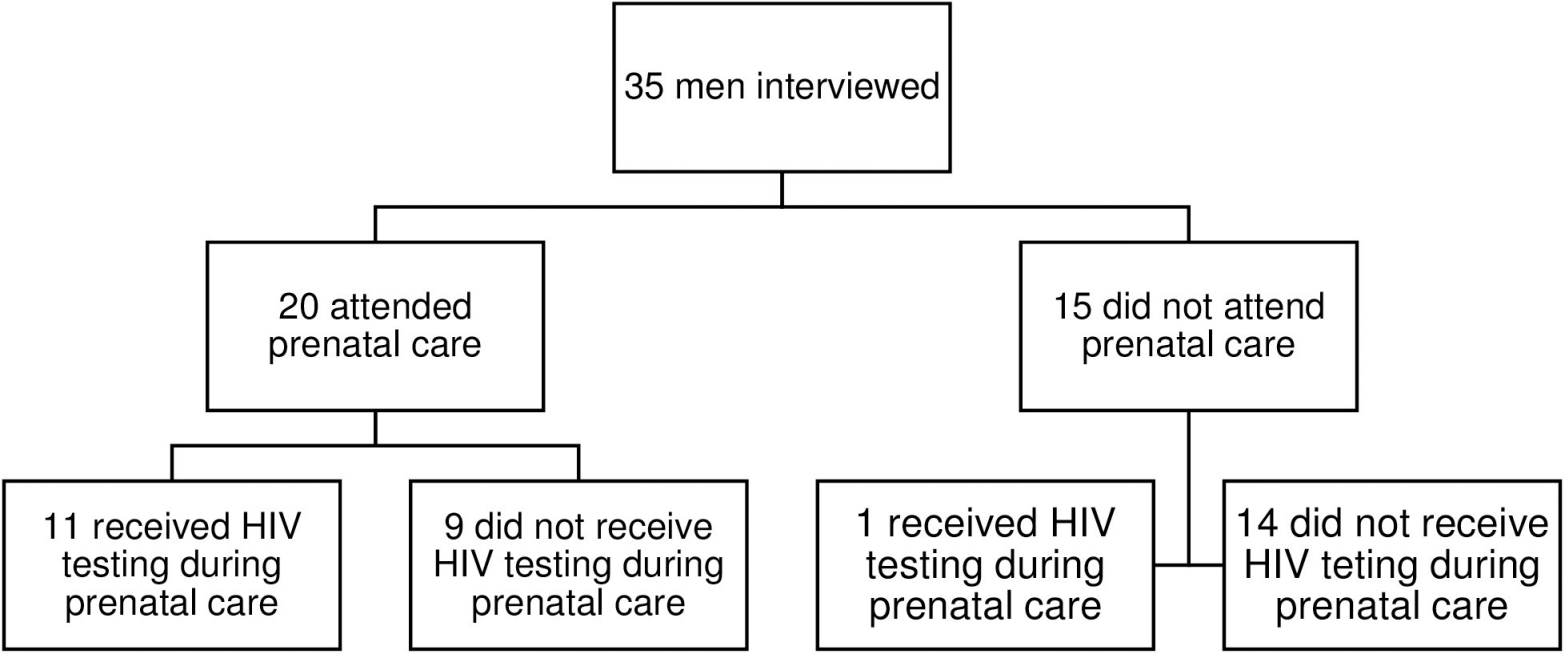
Flowchart of men interviewed regarding participation in prenatal care and acceptance of HIV VCT (voluntary counseling and testing).

**Fig 2:**
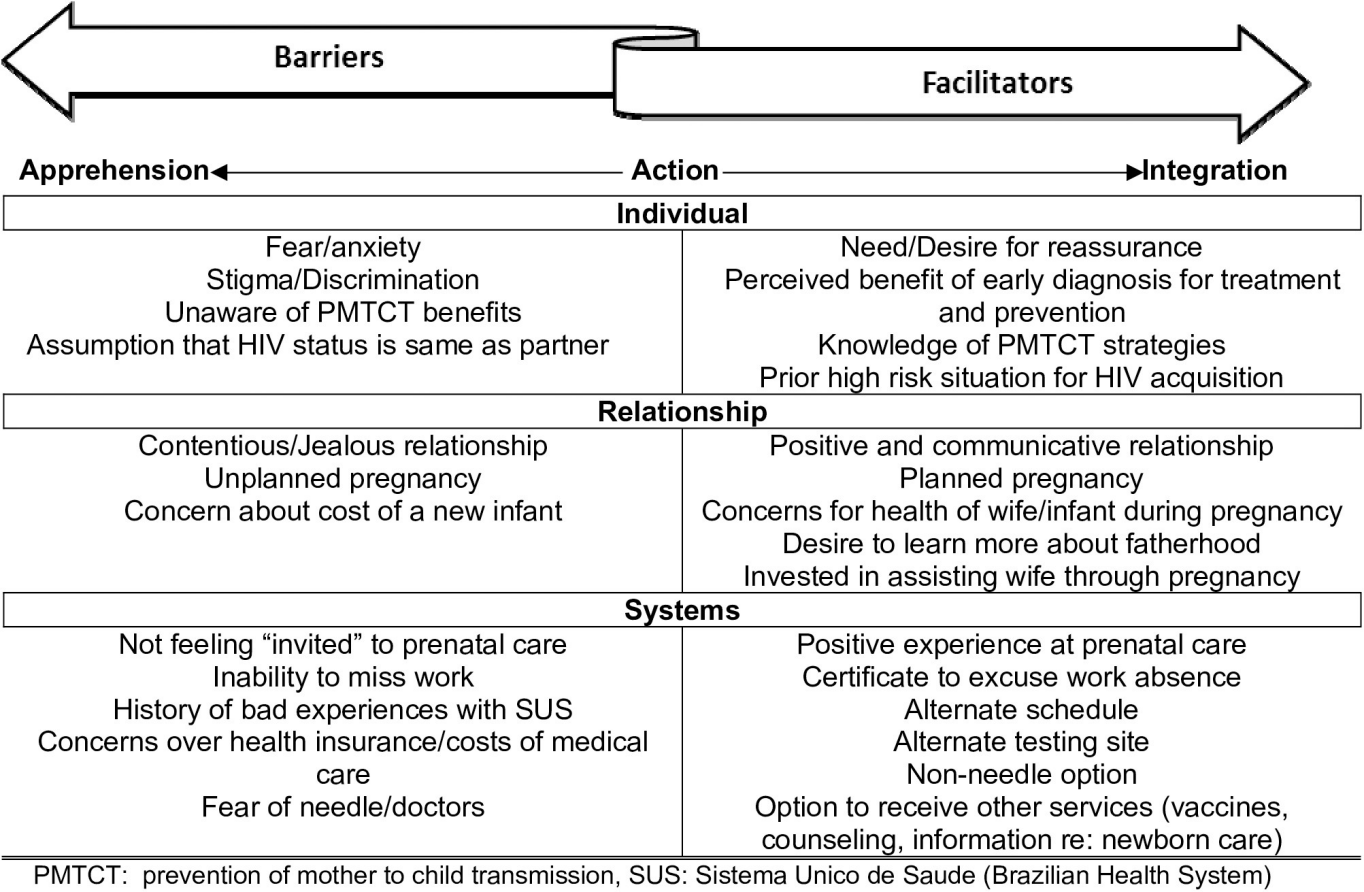
Theoretical model of barriers and facilitators leading to men’s acceptance of HIV testing during prenatal care.

As seen in Fig 1, we interviewed a total of 35 men in the post-partum unit, with roughly half of the participants being non-white. As seen in Table 1, of these 35 men, 20 had attended prenatal care and 11 of them were offered HIV testing. All 11 individuals agreed to HIV testing during prenatal care, and an additional 6 had been tested for HIV prior to partner’s pregnancy. Fifteen men interviewed did not attend prenatal care, and in this group, only 1 was tested for HIV during the partner’s pregnancy and 6 men stated they had ever received HIV testing. None of the participants reported use of injection drugs ever, but 83% reported regular alcohol use, 54% to marijuana use, and 26% to crack cocaine use. As seen in Fig 2, we were able to identify individual, relationship and systemic barriers and facilitators that interacted to determine whether a man participated in prenatal care (integrated) or remained in a state of apprehension by refusing involvement. We found no obvious differences in types of responses based on race in our data. All men stated that if they were offered HIV testing during prenatal care, they would accept.

**Table 1:** General demographics, substance use and HIV testing behavior of men interviewed.

	Attended PN care(n = 20)	Did not attend PNcare (n = 15)	Total: (n = 35)
**Non-white**	10 (50%)	8 (53%)	18 (51%)
**Employed**	17 (85%)	14 (93%)	31 (89%)
**Mean age of men**	28.6 (range 21–37)	27.5 (range 18–43)	28.1 (range 18–43)
**Mean number of children with current partner**	1.3	1.5	1.4
**Mean number of children between two partners**	2.8	2.2	2.5
**Received HIV testing during currentpregnancy**	11 (55%)	1 (7%)	12 (34%)
**Ever received HIV testing**	17 (85%)	6 (40%)	23 (66%)
**Alcohol Use (yes)**	17 (85%)	12 (80%)	29 (83%)
**Crack/Cocaine Use (yes)**	6 (30%)	3 (20%)	9 (26%)
**Marijuana Use (yes)**	11 (55%)	8 (53%)	19 (54%)

### Individual barriers and facilitators

#### Benefits versus negative consequences of HIV testing

Many men stated that they would receive HIV VCT for reassurance of their status since HIV can be “hidden” (Table 2, I1, Table 3 I1-2). Most of the men interviewed were able to identify several benefits to receiving HIV VCT including obtaining appropriate treatment and/or preventing transmission to others (Table 3, I2). Fear and nervousness were identified as the major negative experiences while receiving the testing (Table 2, I1-I2). When asked about how their lives would change if they were diagnosed with HIV, all of the participants stated that they would face a significant amount of prejudice, especially from distant family members and neighbors (Table 2 I4). Although almost all men alluded to discrimination, the men who did not attend prenatal care were more vehement about the negative impacts of being infected, stating that it would affect their relationships with their wife and children, because of their fear of exposing them to the virus (Table 2, I3-4).

**Table 2:** Male partner’s perceived barriers to attending prenatal clinic to receive HIV testing.

INDIVIDUAL	**Fear/Anxiety about HIV status**
	**I.1:** I just was scared, because we never know if we have it [HIV] or not, if we get it or not. What way we could get it. Because it could be hidden. I never had been tested. *(25 yo*, *received HIV VCT during prenatal care)*
	**I.2:** All [testing results] were negative, thank God. My worries were through the roof. *(32 yo*, *received HIV VCT)*
	**Stigma and Discrimination seen against HIV infected individuals**
	**I3:** I know that nowadays it [HIV] is a disease we can live with, but it brings many complications, plus there is all the prejudice..... I don’t know, maybe I would be more resistant to giving care for my child out of fear. I am the do-it-all kind of person, so I always have my hands hurt. I could transmit it. *(43 yo*, *did not attend prenatal care)*
	**I4:** Don’t even joke! Even more when other people know. I know that in my neighborhood there is a man who has it [HIV], and nobody wants to stay close to him. It’s bad. It changes many things. He doesn’t treasure anything.... I can’t tell, but I wouldn’t want to be around anyone if I had HIV… If I knew I had it and she didn’t, I would leave. I couldn’t consider transmitting it to her[wife. *(23 yo*, *received HIV VCT during prenatal care)*
	**Unaware of the increased risk of HIV transmission and acquisition during pregnancy**
	**I5**: I believe the risk [of transmission] is the same; the risk is passing it on to the child. *(33 yo*, *attended prenatal care*, *not offered HIV VCT)*
	**Assumption that man’s HIV status is the same as women’s/children’s status**
	**I6:** They did a lot of tests on her, and if she doesn’t have it I don’t have it, even though I never tested. *(18 yo*, *did not attend prenatal care)*
RELATIONSHIP	**Contentious relationship with arguments about jealousy and money matters**
	**R1**: She is jealous of my own mother, of my family; she says I give more attention to them, and don’t give her. But I don’t know if that is related to the pregnancy, like hormones. It was one of the motives that lead us to split up. *(29 yo*, *did not attend prenatal care)*
	**R2:** I won’t say it’s [relationship] great. We have some arguments because of the jealousy, lots of discussions. We don’t fight in a physical sense, but we have our daily argumen*ts*. *(35 yo*, *did not attend prenatal care)*
	**R3**: She always charged me because of work. That I was going to complete 8 months of unemployment. I didn’t have formal work, but I was doing some gigs. It wasn’t [enough] to support the family, pay the land where we live, it’s hard, even more with the crisis. So when I found work, it got be*tter*. *(18 yo*, *did not attend prenatal care)*
	**Man not involved in planning pregnancy or unplanned pregnancy**
	**R4**: No, none of our kids were planned…Children for me are blessings, they were never punishments. They are gifts from God. He gave me to raise them, they are extensions of what I am; I put 4 seeds that represent that in this world. But now I have to get a vasectomy. *(43 yo*, *did not attend prenatal care)*
	**R5**: I was suspicious, didn’t know if it was true, because we already had a miscarriage, I felt betrayed; our intention was to plan, for her to take care, at first I got surprised, but still was involved a lot; the second time I felt betrayed, there was no way it could be accidental. *(29 yo*, *did not attend prenatal care)*
	**Concerns about money/costs of baby during pregnancy**
	**R6**: I always worry about that [finances]. We started gathering money, opened a bank account, so that nothing would lack at home. We always worry. There is no way of knowing what will happen. Then for any emergency we have some money saved.
	*Interviewer*: *Did you have any concerns over the baby’s health*?
	There are concerns, because we don’t know what will happen, but I was more worried with the financial part than with the health part. *(27 yo*, *did not attend prenatal care)*
SYSTEM	**Not feeling “invited” to prenatal care**
	**S1**: Sometimes the man goes but stays in the waiting room and the mother has the consultation by herself. I went once to a consultation, but I didn’t enter the room. The doctor saw me but didn’t invite me in and I didn’t go. *(26 yo*, *received HIV VCT at prenatal care)*
	**S2:** Just by inviting to take the test. When both parents go to the consultation together, or when the mothers go by themselves, offer a brochure to invite the father. *(32 yo*, *attended prenatal care but not offered HIV VCT)*
	**Inability to miss work because of financial repercussions**
	**S3:** I would go, but we would have to see about the schedule, because I work and I can only miss it if it’s extremely necessary. If she didn’t have any way to go by herself. Because I have bonus payments at work if I arrive on time, if I’m not late. But I’ll lose it if I have to go to the health unit. *(29 yo*, *did not attend prenatal care)*
	**History of prior bad experiences with local health units/Sistema Unico de Saude (Brazilian national Health System)**
	**S4**: Yes, unfortunately SUS [Brazilian national health system] is very treacherous. It is efficient in some cases for example; today that my wife went into labor and was very well handled. I got no complaints there. But for my situation [man has high blood pressure] I do have a complaint, because it has been eight months that I have booked but it has not even been confirmed. I researched it, and it may be eight, nine months, or even a year before I know what has happened to me. I take controlled medicines and my blood pressure has stabilized so I stopped worrying so much, not to the point of exaggeration nor not worrying at all, and that reduced the symptoms I had. *(21 yo*, *received HIV VCT)*
	**S5**: I have nothing against Cuban doctors. However, during consultation, they don`t understand they don`t explain it…We asked two even three times, and she said the same thing, we couldn`t hear the heartbeat because she said the device was broken and had no battery. Anyway, she [Cuban doctor] looked fine, she was there, but she didn`t look like she wanted to be there. *(26 yo*, *received HIV VCT)*
	**Concerns over health insurance/costs**
	**S6:** I was [covered], but it was through private insurance and I wasn’t working, I only had the money for the appointment, not for the exams. *(18 yo*, *did not attend prenatal care*, *diagnosed with gonorrhea*, *but never received HIV VCT)*
	**Lack of non-needle/non-clinic options**
	**S7:** There are a lot of coward men, who are afraid of needles, I think it could help then; you would do it there and then, unless you had to take it to a lab or doctor. But I believe most don’t do it from fear of the results. *(28 yo*, *attended prenatal care*, *but not offered HIV VCT)*
	**S8**: I’m not going to say needles, but of going to the doctor in general, only if I’m really bad. I have a thing with white coats, *(28 yo*, *did not attend prenatal care)*

**Table 3:** Men’s descriptions of perceived facilitators to attending prenatal care to receive HIV voluntary counseling and testing (VCT).

INDIVIDUAL	**Need/Desire for Reassurance**
	**I1:** I thought it [being offered HIV testing] was good, because it’s something you don’t expect. Maybe there is an affair and people will never know until they take it. Because it’s not written in anybody’s forehead, so I believe preventing is the best way. I think everybody should do it. (20 yo, received HIV VCT during prenatal care)
	**To prevent transmission and obtain treatment**
	**I2**: I think it’s important, even if it makes you feel insecure. If you have unprotected sex. It’s important to have control over the situation. You take the test and find out; even for future relationships. Maybe you have a child and don’t transmit it. *(27 yo*, *did not attend prenatal care)*
	**Knowledge of HIV Disease and PMTCT strategies**
	**I3:** It’s an acquired immunodeficiency. It kills the white cells in your body. It doesn’t kill you, but it has symptoms, but other diseases can kill you and the transmission methods are through body fluids. *(29 yo*, *did not attend prenatal care)*
	**I4:** I know the mother can carry the virus, and don’t pass it along to the child, if she takes the medication. I know there is a medication that decreases the odds of transmission to 4%, but it’s not cleared, which is a shame. *(37 yo*, *attended prenatal care*, *was not offered HIV VCT)*
	**Knowing someone with HIV**
	**I5**: I`ve met two of my friends mothers. They both have passed away from HIV. Seems like their husband carried it and they had no idea and they both ended up getting it. *(26 yo*, *received HIV VCT during prenatal care)*
	**History of being in a perceived high risk situation**
	**I6:** When I was a cocaine addict, we always shared the straw, sometimes people had nosebleeds and it got in the straw. Even so we all shared the bloody straw, but I never worried about that back then. *(33 yo*, *prenatal care*, *not offered HIV VCT)*
	**I7:** It was when I got gonorrhea, that I thought I had it. Then one day speaking to a female coworker who is friends with the girl I had sexual relations; and she told me she had HIV, but I thought that she could have been contaminated after our sexual relations. *(18 yo*, *did not attend prenatal care)*
RELATIONSHIP	**Positive and Communicative Relationship with Partner**
	**R1**: There isn’t something I don’t feel comfortable telling her. We get along really well, we share the same ideas. *(24 yo*, *did not attend prenatal care)*
	**R2:** If I said to you that it’s [relationship] perfect, will you believe me? She gave me a boy. Then she gave me my younger girl. She is 2 years old, my sweetheart. And now she gave one more boy. I’m completely crazy about her! *(25 yo*, *received HIV VCT during prenatal care)*
	**Planned pregnancy**
	**R3**: We had it all planned, everything was programmed, and we were about 4 or 5 months in the process of trying, that she has already stopped taking the pill. We were even frustrated, every time her menstrual cycle was late, she would take the test and it would come negative and we got even more frustrated. Then I even started doubting my own ability to conceive a child, because many people told me, even my mom, not to be offensive, but she wondered if I could, because I was married to the other and didn’t have children with her too. Of course we didn’t try, but that still wondered in my head. Then when she was examined, about 5 or 6 months in the process of trying, the result came positive, I had tears falling on my eye, I was so happy. *(32 yo*, *attended prenatal care*, *not offered HIV VCT)*
	**Concerns about the health of the wife/baby during pregnancy**
	**R4:** I was only worried if it would be born healthy, that it didn`t have conditions, like Down syndrome. I wanted it to be born with well-formed organs and all that. *(26 yo*, *received HIV VCT during prenatal care)*
	**R5**: I think only about the Zika virus, because I believe they should have tested it. Because yesterday, coming here, I was talking to a neighbor of mine, mother of some friends who grew up with me. Her son was going to become a father but they did the test and the child had that microcephaly, right? And she was two months pregnant, then the father issued a process, to have an abortion, all legal and right. That is why I came and asked her [his wife] because I thought she had done it. *(32 yo*, *received HIV VCT during prenatal care)*
	**Desire to learn more about being a father**
	**R6:** I think it’s important for the man to be present, to learn stuff even, some things we just don’t know. I am a first time father and I don’t know much about it, I have a sister who is as old as my stepson, but I didn’t live with her… so it’s all new to me. *(22 yo*, *did not attend prenatal care)*
	**Perception that attendance at prenatal care is important as a “father”**
	**R7:** The father who doesn’t attend is an idiot, it’s his child there, there is no reason not to go. *(23 yo*, *received HIV VCT during prenatal care)*
	**R8:** It could be important for the father to be present at those times, which are important for both the baby and the mother. The presence of the father is important then. The father is the father and I think it’s important for the father to attend, *(24 yo*, *did not attend prenatal care)*
	**Invested in assisting wife and/or child through pregnancy since woman is “different”**
	**R9:** I believe that the presence of the man in prenatal care is more psychological related, you show your wife you are there, accompanying her, and she and the pregnancy are important to you. Even more because she will be emotional. Plus the HIV test gives you stability that you are not cheating, didn’t screw up. *(43 yo*, *did not attend prenatal care)*
	**R10:**It should be mandatory, because if the wife is pregnant, the man many times is much more indecent at this time, because the woman is a little bit different. I believe it to be important, especially for the kid. *(34 yo*, *received HIV VCT during prenatal care)*
SYSTEM	**Positive experience receiving HIV test**
	**S1:** Half an hour, just the time to take the test, applied the fingers, drew blood, and it was done…really simple. Very quick and easy. That’s why I think everybody should do it. It doesn’t take long and you get the result there and then. *(20 yo*, *received HIV VCT during prenatal care)*
	**Receiving a “certificate” to excuse work absence or making attendance mandatory**
	**S2**: I think the health units should make it mandatory, but not all mothers have partners, but when the father is present, he would have to take it. It doesn’t have to be all the consultations, but some could be pre established that the father should attend. *(34 yo*, *attended prenatal care but was not offered HIV VCT)*
	**S3:** For the health unit to offer a leave of absence for workers, and also requesting the wife to invite their partners, saying it’s important for the pregnancy. *(27 yo*, *did not attend prenatal care)*
	**Offering HIV VCT at an alternate time**
	**S4:** Many can’t attend at the consultation time, because of work, but an alternative schedule that doesn’t match work time, it can help. *(32 yo*, *attended prenatal care*, *was not offered HIV VCT)*
	**Offering HIV VCT testing at alternate site**
	**S5:** Yes, if they were around a park, and there are some people testing for it. I think that 30 or 40% would stop to get tested, which is a bigger statistic than the one of people who goes to hospitals and health units to get tested. *(28 yo*, *attended prenatal care*, *was not offered HIV VCT)*
	**Non-needle options for testing**
	**S6:** It would make it much easier, I am afraid of needles; if it was done with saliva I would do it every month! *(33 yo*, *attended prenatal care*, *was not offered HIV VCT)*
	**Expand services provided during prenatal care**
	**S7:** I think some orientation would be good, I think mothers come with a natural gift to take care, but men are clumsier. *(29 yo*, *did not attend prenatal care)*
	**S8:** For the man I’d say the HIV and other STDs test, some orientation maybe. You go to prenatal and people stare at you wondering why you are there, it’s not natural. *(28 yo*, *attended prenatal care*, *but not offered HIV VCT)*
	**S9:** I think that along prenatal care there should be a psychological service, the relationship changes a lot during pregnancy, some couples have more fights, financial issues, but the child has nothing to do with that. This should serve both father and mother during prenatal care. *(34 yo*, *attended prenatal care*, *but not offered HIV VCT)*

#### Knowledge of HIV disease

Participants displayed a sophisticated understanding of HIV as a an STI that can diminish the immune system and more than half stated that it could also be transmitted through blood products and shared needles (Table 3, I3-4). Almost all participants accurately described ways to avoid transmission and most men identified treatment that could allow an HIV infected individual to live a long life. In the group of 20 men who attended prenatal care, many of the participants stated that they had friends and/or family members with HIV (Table 3, I5). Men credited the media for educating them about HIV including TV and radio programs.

As regards HIV transmission during pregnancy, although men understood the value of PMTCT (Table 3, I4), none of the men described an increased risk of HIV transmission to both men and women during the time of pregnancy or increased risk of vertical transmission to the infant if the woman acutely seroconverts during pregnancy (Table 2, I5).

There were uncommon examples of misinformation. Three participants who didn’t attend prenatal care stated that they didn’t need to obtain HIV testing as they were unlikely to be infected since their wives and/or children were not infected (Table 2, I6).

#### HIV self-risk assessment

None of the participants thought they were currently at risk for acquiring HIV, but several admitted that there were times in the past that they were concerned about exposure to HIV due to sexual acts or drug use with possibly infected individuals (Table 3, I6-7).

### Relationship facilitators and barriers

#### Feelings towards wife/relationship

Almost all of the men interviewed described their relationship with the mother of their infant using positive language, ranging from “comfortable” to “perfection” (Table 3, R1-2). A few admitted that their relationship during pregnancy faced difficulties, with two men who did not attend prenatal care describing frequent relationship break ups (Table 2, R1-2). All men stated that they have arguments with their partners with “jealousy” being the most frequently cited cause of contention (Table 2, R1-2), followed by financial worries (Table 2, R3).

#### Feelings towards fatherhood/pregnancy

Approximately half of participants described their pregnancy as planned and described great joy in finding out the news, including several men stating that they cried out of happiness (Table 3, R3). The remaining half of the men interviewed stated that they had not planned on having another child (Table 2, R4-5). Despite these men describing great amounts of surprise and shock at the news, all but one of the participants described a high level of enthusiasm for the pregnancy, stating that they were happy once the shock faded (Table 2, R4). Of note, most of the fathers who attended prenatal care had a planned pregnancy versus only a few of the fathers who did not attend prenatal care.

#### Infant worries

When men were questioned about any concerns they felt during the pregnancy, men who attended prenatal care identified health concerns they had for the mother and/or baby (Table 3, R4-5). Two participants who attended prenatal care mentioned Zika virus, one identified concerns about toxoplasmosis, but most had general concerns about making sure their baby was healthy given personal or familial experiences with difficult pregnancies or poor neonatal outcomes. Interestingly, men who did not attend prenatal care stated that they had concerns about financially supporting their infant, and this concern seemed to outweigh health concerns (Table 2, R6).

#### Motivation to attend prenatal care

There were three categories of responses about which factors motivated men’s participation in prenatal care. Some men thought male participation was important to learn about being a father (Table 3, R6). Others attended prenatal care because they felt that it was their duty as the father and/or man, because “the father is the father” (Table 3, R7-8). Others emphasized that the woman is more vulnerable during pregnancy, using words like “different,”“emotional,” “forgetful” and that because of this increased vulnerability, she needs the man’s help to optimize their child’s health (Table 3, R9-10). Several mentioned that because the woman is “different” men may have outside sexual relationships (Table 3, R10).

### Systems based barriers and facilitators

#### Financial concerns affecting medical seeking behavior

Most of the men we interviewed stated that they were currently employed with a majority in the service industry (doorman, restaurant worker, etc.), and all men stated that they had a support system including family members, friends and/or their church that could provide them with financial assistant if needed. Most stated that they had intermittently asked for financial help, but that they were not dependent on these sources for income.

Almost all men received prior medical care, with most men describing a medical encounter for an urgent issue. Only six men (17%) sought preventative medical care because their place of work required a medical examination. Several men endorsed being diagnosed with STIs with three (8.5%) individuals having prior diagnoses of syphilis, one (3%) man with a prior diagnosis of gonorrhea, one (3%) man diagnosed with HSV and two (6%) men mentioned an undefined disease they got from their wives that required treatment. Three participants who attended prenatal care complained about the national health care system, the Sistema Único de Saúde (SUS), for not providing adequate health services citing long wait times and lack of appropriate doctors, although all agreed that the labor and delivery services provided by Hospital Conceiçao were adequate and even commendable (Table 2, S4-5).

#### Convenience of testing

Of those who took the test, all of them described the actual testing process as quick and easy, lasting anywhere from four minutes to half an hour (Table 3, S1). Scheduling of the exam seemed to be the most profound barrier to attending prenatal care to obtain testing as most stated that they had work during clinic hours, and it would be financially costly for them to take time off (Table 2, S3).

Participants stated that they did NOT feel “invited” to prenatal care (Table 2, S1-2). Nine men who attended prenatal care did not receive an invitation for HIV VCT. Most men suggested that if they were invited either with a phone call from the health center, by the pregnant woman on behalf of health center, or with a paper invitation/certificate they could show their job supervisor, they would be more motivated to attend (Table 3, S2-3).

#### Improvements to prenatal care

Other systematic improvements discussed by the men included using non-needle tests and a non-clinic testing center (Table 3, S4-6). These issues were of variable importance with some men stating that their personal fear of needles and/or doctors was a notable barrier to testing (Table 2, S8) and several men suggesting that this fear may actually be an “excuse” covering deeper fears of actually being HIV infected (Table 2, S7).

Participants also suggested a variety of services they would appreciate receiving during prenatal care, ranging from vaccines and STI testing to couples counseling and complete physical exams. Most commonly, men requested an orientation to newborn care, including how to bathe them, change their diapers and feed them (Table 3, S7-9).

## Discussion

Few if any studies have evaluated perceived barriers and facilitators to HIV VCT among male partners of pregnant women in South America. Our participants did not suggest that attending prenatal care was only a woman’s domain or “unmanly”, as has been described in African countries.[4,31]. In fact, most of the men signaled that communication in the partnership was open and easy, and they would respond well to the woman issuing an invitation to attend prenatal care.

Other studies also show that misconceptions about HIV infection plays a notable role in men’s refusal to receive testing, including men’s assumption that their partner’s HIV status is a proxy for their own status.[11,15,32,33] Furthermore, men in these prior studies did not know about the PMTCT benefits of HIV testing. [4,9] In Brazil, participants appeared to have a very nuanced and detailed understanding of HIV infection from the ongoing media campaigns. Three men did mention that they did not need HIV testing since their partner and/or child was HIV negative, but in general, men seemed to think that testing was important to “remove any doubts” and to obtain appropriate treatment and preventative services, including PMTCT. Men were not aware that in some studies, pregnancy has been shown to increase the risk of HIV acquisition in both men and women [34–37], nor were they aware of the increased risk of HIV vertical transmission to infants with acute seroconversion during pregnancy.[38–40] Future attempts to educate men and women should focus on the vulnerability of all members of the family unit to HIV infection during pregnancy.

Our study did echo one major “system-wide” finding from previous research as many of the men implied that the prenatal care clinic was not inviting. Several men said they sat in the waiting room, but there was no effort to include them in the visit, which is an unfortunate missed opportunity that must be addressed. Similar to other studies where men were reluctant to learn of their own status as it is “mental torture” to be positive, [4] men in our study described being diagnosed with HIV would result in stigma and discrimination. This sentiment was even more pronounced in those who did not attend prenatal care, either because this fear deterred men from prenatal care or because men who attended prenatal care received more HIV related counseling and reassurance. When men were asked about concerns they had about their new infants, men who did not attend prenatal care focused more on the financial burdens of an expanding family, whereas those who attended prenatal care were more focused on the health of the child. Furthermore, men who planned their pregnancy were more involved with prenatal care. This may suggest that since the pregnancy was planned, they were more engaged as well as emotionally and financially prepared, thus able to take time off from work and other commitments to participate in care of the infant and woman.

Despite having a comprehensive national health plan, most Brazilian men interviewed are still not receiving preventative services unless it is mandated by their place of work. There appears to be a general sense of dissatisfaction with the Brazilian government inclusive of the national health system with several men who attended prenatal care describing long wait times to receive appropriate care and perceived unprofessional behavior of health care providers. At least two men suggested that the government was “hiding” information about Zika virus with several men naming “Dilma” aka Dilma Rousseff, ex-president of Brazil, as a source of discontent. This distrust of the medical system likely translates into lower likelihood of accessing medical services including HIV testing. If attendance is optimized, prenatal care can serve as an important entry point to diagnose and treat multiple diseases including HIV, syphilis, and Hepatitis C; all diseases with high prevalence rates in the south of Brazil with available treatment and important transmission consequences to pregnant women and infants.

Our conceptual model was derived from the HIV Testing Decisions Model,[29] but modified to reflect the perspectives offered during these in-depth interviews. The initial conceptual framework was developed based on a study performed on men who have sex with men (MSM), intravenous drug users and clients of an STI clinic in Seattle, Washington. Although “individual” factors were similar in both populations including stigma, risk perception, and participant’s understanding of the benefits of early testing/treatment, in our particular population, “relationship” factors including the strength of the partnership and men’s involvement as a father were equally important to motivate or deter men’s participation in prenatal care for HIV testing. We also combined the system and counseling/testing barriers from the original model as there was notable overlap between the two, with a strong emphasis on men wanting recognition of their importance to prenatal care, with men requesting clinic-based invitations, excuses from work, and additional services tailored towards them. Interestingly, some of the themes relayed by the men echoed our previous research at this site, where we questioned women whose partners did not participate in HIV testing. During our two previous studies at Hospital Conceiçao, more than half of women indicated that inconvenience (due to work/distance) was by far the most common reason for non-participation.[22,23] Other reasons included low self-perception of risk, testing not being important, and being afraid of test results. In contrast to this study, only one woman directly referred to the prejudice faced by HIV positive individuals and none commented on the state of their partnership or the man’s perceived role as a father, whereas in this study, men’s perception of fatherhood and desire to improve their newborn’s health seemed to be great motivators for participation.

This study had several limitations. We used a female interviewer because she was the best trained individual with extensive experience performing in-depth interviews on sensitive topics, but we recognize that there may be a gender bias discouraging some men to openly discuss socially unacceptable behavior with a woman. This bias seemed minimal based on review of audiotaped results. Moreover, this was a cross-sectional qualitative study thus we were not able to show correlation between various predictors and the outcome of increased attendance in prenatal care. However, it is the first study performed in South America that provides a detailed description of facilitators and barriers in involvement of male partners of pregnant women in prenatal care. Furthermore, our sample population is an especially at risk group of individuals, as evidenced by the high rates of drug and alcohol use reported, which are especially correlated with HIV acquisition risk in Porto Alegre [41]. Cross-sectional studies in Porto Alegre evaluating high risk populations also have noted similar drug use results.[42] The generalizability of our findings are limited given our purposive approach to sampling an equal number of non-white men and the small number of men interviewed. We also only interviewed men who were visiting their newborns, which may bias our results to fathers who are more invested in their child. Of note, our clinicians and hospital epidemiologist at the site stated that per their estimates, >85% of men visit their newborn in the hospital. Finally, we only interviewed men at a public facility, mainly because partner testing is specifically recommended and provided free of charge in this consortium of hospitals. It was not a widespread recommendation for all clinics in the private sector.

## Conclusion

Our study agrees with previous work [6,9] that external barriers are the most significant deterrent to receiving HIV testing. Almost all men suggested if invited by the health center with an excuse from work via a certificate/mandate, they would attend prenatal care and obtain HIV testing. Our current approach of making pregnant women responsible for involving their partners is inadequate. Future efforts should reorient prenatal care towards serving the entire family by using couples oriented counseling methods [12,43] with a clear focus on protecting the infant from preventable diseases. Interventions should include issuing a formal invitation to the father of the infant, offering weekend and/or evening hours, considering mobile vans and offering non-needle test options to optimally access this more difficult to reach population.

## Abbreviations

PMTCT: Prevention of Mother to Child Transmission
HIV: Human Immunodeficiency Virus
VCT: Voluntary Counseling and Testing
STI: Sexually Transmitted Infections
SUS: Sistema Unico de Saude (Brazilian Health System)
